# Purification and characterisation of the extracellular cholesterol oxidase enzyme from *Enterococcus hirae*

**DOI:** 10.1186/s12866-015-0517-2

**Published:** 2015-09-14

**Authors:** Hany M. Yehia, Wesam A. Hassanein, Shimaa M. Ibraheim

**Affiliations:** Food Science and Nutrition Department, College of Food and Agriculture Sciences, King Saud University, Riyadh, Saudi Arabia; Food Science and Nutrition, Faculty of Home Economics, Helwan University, Helwan, Egypt; Department of Botany (Microbiology), Faculty of Science, Zagazig University, Zagazig, Egypt

**Keywords:** Cholesterol oxidase enzyme (ChoX), *Enterococcus hirae*, Decomposition, Food samples, ChoX molecular weight

## Abstract

**Background:**

Recently many efforts are being carried out to reduce cholesterol in foods. Out of the 50 selected isolates that were tested using the agar well diffusion method to assess their ability to decompose cholesterol, 24 bacterial isolates were screened based on their cholesterol-decomposition ability in liquid media.

**Results:**

The bacterial isolate that displayed the highest cholesterol oxidase activity was identified as *Enterococcus hirae*. The maximal growth and cholesterol decomposition were achieved with a 1-day incubation under static conditions at 37 °C in cholesterol basal medium adjusted to pH 7 supplemented with 1 g/l cholesterol as the substrate, no additional carbon or nitrogen sources and 0.5 % CaSO_4_. The cholesterol oxidase enzyme (ChoX) produced by *E. hirae* was extracted at an (NH_4_)_2_SO_4_ saturation level of 80 % and purified with 79 % yield, resulting in 2.3-fold purification. The molecular weight of (ChoX) was 60 kDa. The optimal conditions required for the maximal activity of the purified COD enzyme produced by *E. hirae* were 30 min, 40 °C, pH 7.8, substrate concentration of 1 g/l and 200 ppm of MgCl_2_. The enzyme maintained approximately 36 % and 58.5 % of its activity after 18 days of storage at 4–8 °C. Also, the enzyme loss its activity by gradual thermal treatment, but it maintained 58.5 % of its activity at 95 °C for 2 hr.

**Conclusions:**

*E. hirae* Mil-31 isolated from milk had a great capacity to decompose cholesterol in basal medium supplemented with cholesterol under its optimal growth conditions. Decomposition process of cholesterol by this strain results from its production of cholesterol oxidase enzyme (ChoX). The highest specific enzyme activity and highest purification fold of purified enzyme were achieved after using Sephadex G-100.

## Background

Cholesterol is a waxy, fat-like substance that is found in all cells of the body. Cholesterol enters the human body mainly through food [1], and the majority of the cholesterol in the body originates from the liver [2]. Cholesterol is required for the formation of sex hormones [3], Cholesterol can also be converted to bile acids in the liver and vitamin D in the skin and kidney [4] and the formation of bile acids that help the body to digest fat [5]. Many studies have reported the ability of different bacteria to reduce the cholesterol levels in aqueous systems, such as liquid media [6] and the blood serum [7].

Some bacteria not only utilize cholesterol as a sole carbon source [8] but also decompose cholesterol via the cholesterol oxidase enzyme (ChoX) and produce different intermediate compounds [9]. *Enterococcus faecium* CX and *Lactobacillus acidophilus* N5, which colonise the intestinal tract and survive under gastric conditions, assimilate cholesterol and reduce its level in serum [10]. Pereira and Gibson (2002) [11] found that *Lactobacillus fermentum* strains F53 and KC5b, *Bifidobacterium infantis* ATCC 15697, *Enterococcus durans* DSM 20633, *Enterococcus gallinarum*, and *Enterococcus faecalis* have *in vitro* cholesterol-reducing abilities [11].

A variety of ChoX enzyme producing microorganisms have been isolated by [12], most of them produce cell-bound ChoX enzyme, but some Actinomycetes are able to produce high levels of extracellular ChoX enzyme, which is generally easier for isolation and purification than intracellular enzymes [12]. *Streptomyces* sp. produce COD enzyme extracellularly [13] and the corresponding structure gene for COD enzyme has been cloned and sequenced from different *Streptomyces* as *Streptomyces fradiae* [14], *Streptomyces natalensis* [15]. *Gamma-proteobacterium* Y-134 [16], *Burkholderia cepacia* [17], *Cellulomonas* [18], *Brevibacterium sterolicum* [19], *Brevibacterium sp.* [20], also produce extracellular COD enzyme.

Cholesterol degrading bacteria from cow’s milk and about 11 bacterial isolates exhibited cholesterol degrading activity with cholesterol lowering potentials ranging from 42.88 –97.20 % [21]. The ChoX enzyme from *Burkholderia cepacia* strain ST-200 produces 6-beta-hydroperoxycholest-4-en-3-one from cholesterol [22]. The oxidation of cholesterol produces the intermediate 3-ketosteroid as the final product [23]. Cholesta-4, 6-dien-3-ol, cholesta-4, 6-dien-3-one, and cholesta-3, 5-dien-7-one are produced from the oxidation of cholesterol [9]. Doukyu and Aono (2001) [22] reported that the ChoX enzyme from *Burkholderia cepacia* strain ST-200 produces 6-beta-hydroperoxycholest-4-en-3-one from cholesterol. The oxidation of cholesterol produces the intermediate 3-ketosteroid as the final product [23]. Liu and Shan (2006) [9] reported that cholesta-4,6-dien-3-ol, cholesta-4,6-dien-3-one, and cholesta-3,5-dien-7-one are produced through the oxidation of cholesterol. Numerous factors affecting the activity of the cholesterol oxidase enzyme have been studied by many different research groups [24, 25].

The optimisation of culture conditions is an important consideration when studying cholesterol oxidase enzymes in bacteria. In particular, the culture conditions influence the properties of the enzyme [25, 26].

Purification and characterisation of several microbial cholesterol oxidase enzymes were conducted by [14, 24]. Niwas et al. (2014) [27] suggested that calcium alginate entrapment is a promising method for the immobilisation of *Streptomyces* sp. and also suggested that the immobilized cells may produce ChoX at higher quantities after three consecutive fermentation cycles compared with free cells. The results obtained can be exploited for commercial purposes. Lashkarian et al. (2010) [28], were instructed a recombinant plasmid containing ChoX gene from *Rhodococcus* sp. strain 50, cloned into cloning vector (STV28) and transformed into *E. coli* strain DH5α to produce a significant levels of extracellular ChoX in an optimized medium for a short period.

The importance of the ChoX enzyme is derived from the biotechnological purposes for which the enzyme is applied. This enzyme is industrially important for the production of pharmaceutical steroids [29], determination of cholesterol in food by coupling with peroxides [30], determination of the total cholesterol in several real food samples such as eggs and meat [31], and in agriculture for insecticides [32]. This ChoX enzyme is widely used for bioconversions for the clinical determination of blood serum cholesterol [30].

The objectives of this study are the enumeration and isolation of cholesterol-decomposing bacteria from different sources, the screening of isolates based on their ability to decompose cholesterol in liquid media, the optimisation of some growth conditions for maximal cholesterol decomposition, and the production, purification and characterisation of the ChoX enzyme produced by the most active isolates.

## Methods

### Enumeration and isolation of cholesterol decomposing bacteria

Different food samples (*n* =100) from many sources were collected from local markets located in Zagazig city located in Egypt. 20 samples of each product were collected (cheese, yogurt, raw milk, whey and milk). The spread plate technique [33] and basal medium [34] were used. The Medium was prepared as follows (g/l): NH_4_Cl, 0.5; NaCl, 0.5; KH_2_PO_4_, 0.4; K_2_HPO_4_, 0.3; MgSO_4_, 0.2; yeast extract, 0.1; agar 20 g; and distilled water up to 1000 ml, pH 7 ± 0.2. Cholesterol was added at a concentration of 1 g/l [35]. The media did not contain a carbon source (cholesterol), but it was sterilised separately. Cholesterol was emulsified according to method described by Imshenetskii et al. (1968) [36], where 1 g of cholesterol was dissolved in 35 ml of boiling acetone. Then added to 200 ml of distilled water heated to 90–95 °C drop-by-drop. Then it was filtered, condensed under vacuum to remove the acetone. The resulting cholesterol emulsion was sterilized at 0.5 atm. for 30 min [37] then the solution was stored in a refrigerator. The ability of bacteria to decompose cholesterol was estimated by the appearance of clear zones of translucency around colonies on agar medium after incubation at 37 °C for 24 h [37]. The numbers of cholesterol decomposing bacteria were determined.

### Screening of bacterial isolates for cholesterol decomposition

- Well diffusion agar method:
  All bacterial isolates were purified and maintained in the basal media and tested for cholesterol decomposition using the well diffusion agar method as described [38].
- In liquid medium:
  The experiment was carried out to select the most potent bacterial isolates that have the high percent of cholesterol decomposition added in their liquid medium. Cholesterol was quantitatively measured using a colourimetric method (Liebrman-Burchard reaction [39]. The residual cholesterol was calculated according to the equation of [17] as follows:
  Residual cholesterol (mg/dl) = Absorption of standard/Absorption of standard × Conc. of standard.
  The amount of decomposed cholesterol (mg/dl) = Amount of cholesterol in control – Amount of residual cholesterol of sample.
  The % of cholesterol decomposition = Amount of decomposed cholesterol/Amount of cholesterol in control × 100.

### Identification of the most abundant producer bacterial isolates

The most active ChoX enzyme producer isolate was identified using a Biolog Microlog 34.20 system at the Unit of Identification of Microorganisms and the Biological Control Unit of the Agriculture Research Centre, Giza, Egypt.

### Some factors affecting the growth and decomposition of cholesterol by the tested bacteria in liquid media

For studying the factors affecting the growth of bacterial strain and decomposition of cholesterol, constant condition was used throughout the study as follows: conical flasks (250 ml in volume) containing 100 ml of fermentation broth basal medium [34] and contained 0.1 g cholesterol were inoculated with one ml of standard inoculum of the bacterial strain cultures and incubated at 37 °C for 24 h. Some growth factors affecting cholesterol decomposition were studied, such as different incubation temperatures (20 to 50 °C) and incubation periods (1 to 3 days); different pH values using acetate buffer (pH 3.6 up to 5.6) and phosphate buffer (pH 6 up to 8) to adjust the pH; static and shaking conditions at different speeds (80 to 140 rpm); different concentrations of cholesterol such as 0.5 to 2 (g/l); different carbon sources (glucose, fructose, sucrose, maltose, lactose, galactose and glycerol) at a final concentration of the carbon source 1 % w/v [40]; organic nitrogen sources (peptone, yeast extract and beef extract) at a final concentration of medium 0.5 %, w/v [40]. Inorganic nitrogen sources (potassium nitrate, sodium nitrate and ammonium sulphate) at a final concentration equimolecular to that in 3 g of NaNO_3_, different concentrations of metals (BaCl_2_, MnCl_2_, CaSO_4_, Na_2_HpO_4,_ K_2_HpO_4_ and MgSO_4_) and different growth media (nutrient, yeast and basal medium) were also studied. The bacterial strain was grown at different parameters then cholesterol decomposition assay was performed.

### ChoX enzyme assay

The activity of the extracellular enzyme was determined according to the method described by Inouye et al. (1982) [41], as follows: to 0.4 ml of 125 mM Tris-HCl buffer pH 7.5, 0.1 ml of cell free extract was added, and the mixture was incubated in water bath at 37 °C. After 3 min. 25 μl of 12 mM of cholesterol in isopropanol solution were added to the mixture, then after 30 min., 2.5 ml of absolute ethanol were added to the reaction medium and the amount of 4-cholesten-3-one was determined by measurement of the absorbance at 240 nm. Reaction blanks were prepared by replacing 25 μl of cholesterol solution for 25 μl of isopropanol. Then ChoX activity (U/ml) and ChoX specific activity (U/mg. protein) were calculated.

One unit of cholesterol oxidase activity (U): was defined as that which brings about the formation of 1 μmol of 4-cholesten-3-one in 30 minutes at 37 °C.

### Protein estimation

Protein was estimated by the method of Lowry et al. (1951) [42], using bovine serum albumin (10 – 100 μg/ml) as a standard measured at 750 nm.

### Purification of ChoX enzyme

Ammonium sulphate was added to the culture filtrate up to 80 % saturation for the partial purification of the enzyme. The precipitate was dissolved in 10 mM 0.2 M Tris-HCl buffer, pH 8. The precipitate was then dialysed against the same buffer. This preparation was applied to a Sephadex G-100 gel chromatography column. Fifty fractions (each containing 5 ml) were collected at 1 ml/min. Both enzyme activity and protein content were determined for each separate fraction. The molecular weight of the purified cholesterol oxidase enzyme produced by *E. hirae* was determined by sodium dodecyl sulphate polyacrylamide gel electrophoresis (SDS–PAGE), which was performed according to the method [43], as modified [44] at the Agriculture Genetic Engineering Research Institute (AGERI), Giza, Egypt.

### Factors affecting the activity of purified ChoX enzyme

The effect of different concentrations of enzyme (0.1 to 0.7 mg/ml) at different incubation periods (10 to 90 min); different incubation temperatures (20 to 55 °C); different pH values (7.2 to 9.0) by adjusting pH with 0.2 M Tris-HCl buffer); different cholesterol concentrations (0.25 to 1.75 (g/l)); different metal ions (EDTA, cadmium chloride, manganese chloride, potassium chloride and cobalt chloride) at different concentrations (100 to 300 PPM); and different storage periods (2 to 18 days) were estimated (1 ml of cholesterol emulsion (1 g/l) added to different concentrations of enzyme (0.29 to 1.45 mg protein/ml) were incubated for 30 min at 37 °C. The enzyme activity at each concentration was determined as previously mentioned). Additionally, the thermal stability of purified ChoX was studied at 45 to 95 °C for 2 h.

The obtained data were statistically analysed to determine the standard deviation, and differences between means were assessed as described [45]. Bivariate correlation matrix analysis of the obtained data was performed using SPSS software program (Version 8) as described [46].

## Results and discussion

Cholesterol decomposing bacteria were enumerated and isolated from different food samples (*n* = 20 for each) namely yogurt, whey, milk, cheese and raw milk using basal cholesterol medium and dilution method. It was obvious from the obtained results in Table 1, that there were no noticeable differences in the counts of these microorganisms in the tested samples. Also it was found that the highest bacterial count was found in yogurt (Log N = 6.4), followed by raw milk and milk (values of 6.38 and 6.37, respectively) while the lowest bacterial count was represented in cheese and whey (6.35 and 6.28 respectively. Fifty bacterial isolates were screened for cholesterol decomposition on basal cholesterol agar medium using the well diffusion agar method. Out of 50 cholesterol decomposing isolates, only 24 bacterial isolates were selected for further screening due to their decomposition of cholesterol added to their liquid media. The highest percentages of cholesterol decomposition (75.3, 69.8, 60.08 and 54.8 %) were recorded for bacterial isolates M-31, Ch-14, Y-28 and W-42, respectively. The mean percent cover value for the five foods were analysed by Duncan’s multiple range test. This test revealed that mean within the column was not significantly different due to the difference among bacterial.

**Table 1:** Screening of bacterial isolates for cholesterol (100 mg/dl) decomposition in liquid medium and total viable count on solid medium (Log N)

Isolate source	Isolate No.	Residual cholesterol (mg/dl)	% of decomposition	Dry weight (mg/50 ml)	Log N	Total isolates
Raw milk (RM) (*n* = 20)	1	64.80	35.2	13.3	6.38	7
	30	84.86	15.14	5.70		
	36	69.89	30.11	11.4		
	38	49.74	50.26	19.02		
	50	59.80	40.2	15.22		
Mean SD		^*^65.82^a^	^*^34.18^a^	^*^12.92^a^		
		12.98	12.99	4.92		
Whey (W) (*n* = 20)	2	84.90	15.10	5.70	6.28	14
	8	90.30	9.70	3.80		
	16	59.72	40.28	15.20		
	24	84.86	15.14	5.70		
	42	45.20	54.80	20.93		
Mean SD		^*^72.99^a^	^*^27.00^a^	^*^10.26^a^		
		19.65	19.56	7.44		
Yogur (Y) (*n* = 20)	3	74.86	25.14	9.50	6.40	9
	10	64.89	35.11	13.30		
	18	64.39	35.61	13.30		
	28	39.92	60.08	22.82		
	44	79.73	20.27	7.60		
Mean SD		^*^64.75^*a^	^*^35.24^a^	13.30^a^		
		15.35	15.35	5.86		
Milk (M) (*n* = 20)	4	69.75	30.25	11.40	6.37	9
	7	49.70	50.30	19.00		
	22	54.63	45.37	17.12		
	31	24.70	75.30	28.54		
Mean SD		^*^49.69^a^	^*^50.30^a^	^*^19.01^a^		
		18.72	18.72	7.10		
Cheese (Ch) (*n* = 20)	12	79.50	20.50	7.60	6.35	11
	14	30.20	69.80	26.63		
	34	54.75	45.25	17.12		
	40	75.13	24.87	9.51		
	46	54.66	45.34	17.12		
Mean SD	24	^*^58.84^a^	^*^41.15^a^	^*^15.59^a^	^*^6.35^a^ 0.041	50
		19.66	19.66	7.53		

*n*=20 : is the number of each sample

^*, a^Different superscripted letters indicate that no significant differences (*P* ≤ 0.05) among the observed values within columns (Duncan’s multiple range test)

**Fig. 1:**
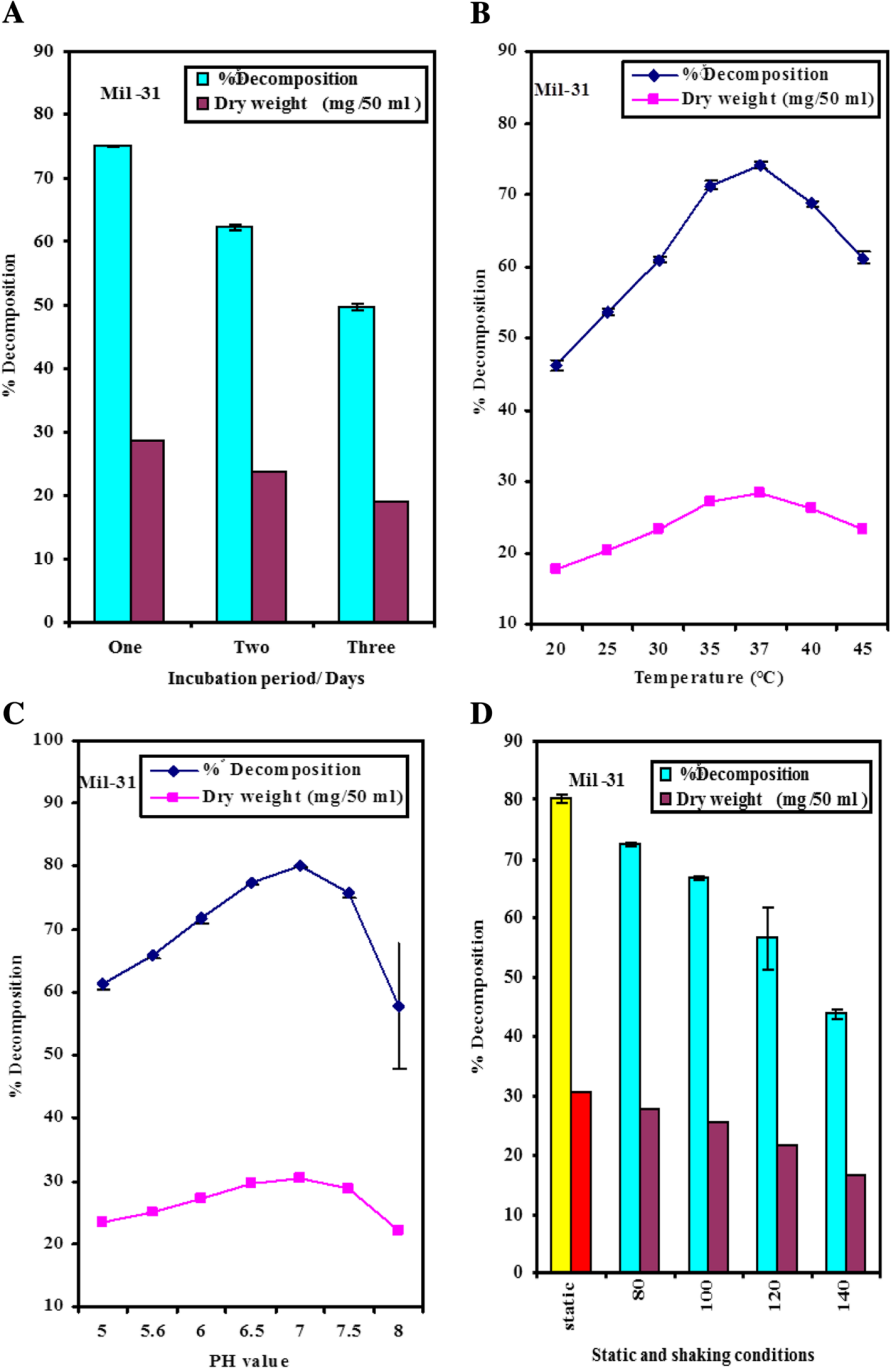
(**a**, **b**, **c** and **d**) Effect of different Incubation periods, temperatures, pH value, static and shaking conditions on the biomass and cholesterol decomposition by *E. hirae* respectively. Difference between values is statistically significance level of 0.05

The highly active cholesterol-decomposing isolate M-31 was selected and identified as *Enterococcus hirae* using a Biolog Microlog 34.20 System at the Unit of Microorganisms, Identification of Microorganisms and Biological Control Unit of the Agriculture Research Center, Giza, Egypt. In this study, different factors affecting the growth and cholesterol decomposition by *E. hirae* were studied. The results shown in Fig. 1a , demonstrate that the maximal cholesterol decomposition (75.1 %) in liquid medium was achieved after one day. The results presented in Fig. 1b show that the maximal percentage of cholesterol decomposition (74.2 %) in liquid medium by *E. hirae* was obtained at 37 °C. Above and below this particular incubation time there was a reduction in the ability of tested bacteria to cholesterol decomposition. Food is considered a good source of cholesterol decomposing microorganisms. Pathogenic *mycobacteria* are able to take up, modify, and accumulate cholesterol from liquid growth media and form a zone of clearance aound a colony when plated on solid media containing cholesterol [47]. The reduction of cholesterol levels *in vitro* or *in vivo* by microorganisms may take place via non-enzymatic [12] or enzymatic processes [8]. The latter bacteria may decompose cholesterol due to their production of extracellular cholesterol oxidase (ChoX) enzyme. These results are in agreement with [48], who reported that some intestinal bacteria such as *Bifidobacterium, Eubacterium, Lactobacillus, Enterobacteriaceae, Clostridium* and *Enterococcus* decompose cholesterol via the ChoX enzyme to cholest-4-en-3-one. Additionally [13], reported that cholesterol might be completely oxidised by microbial ChoX to generate carbon dioxide and water. Moreover, *Cellulomonas* [18], *Brevibacterium sterolicum* [28], *Streptomyces natalensis* [15] and *Brevibacterium* sp. [20] are ChoX -producing bacteria.

Environmental and nutritional factors are known to influence the growth and decomposition of cholesterol by microorganisms. The optimisation of these culture conditions was achieved by Chang and Chou (2002) [49] and Kim et al. (2003) [25]. The optimum temperature for the decomposition of cholesterol in liquid medium by *Brevibacterium* sp. was 37 °C [13]. In contrast, several studies have reported other optimal incubation temperatures, such as 34 °C for *Streptomyces fradiae* [14], 29 °C for *Rhodococcus erythropolis* ATCC 25544 [24] and 30 °C for *Bacillus subtilis* SFF34 [28].

Moreover, the results in Fig. 1c, show that pH 7.0 was the optimum pH value for cholesterol decomposition (80.2 %) in liquid medium by *E. hirae*. It was found that, optimal pH value required for attaining maximum growth and decomposition of cholesterol by *E. hirae* was pH 7.0 using 0.2 M acetate buffer and 0.2 M phosphate buffer.

In contrast, the pH value of the culture medium plays a critical role in the optimal physiological performance of the cells and the transport of various nutrient components across the cell membrane, and the decomposition of cholesterol is affected by a change in the pH value of the media. It was previously reported that the optimal pH values for cholesterol decomposition are 7.2 for *Streptomyces fradiae* [14] and 6.75 for *Rhodococcus erythropolis* ATCC 25544 [24].

The results in Fig. 1d, show that static conditions were better than shaking conditions for the decomposition of cholesterol by the tested strain in the liquid media. Additionally, the results indicate that the highest percentage of cholesterol decomposition by *E. hirae* was (80.1 %). *E. hirae* favored static condition for growth and decomposition of cholesterol than shaking conditions. Although shaking conditions are generally better than static conditions for the optimal cholesterol decomposition of aerobic bacteria [14, 50], this doesn’t apply for the facultative anaerobic bacteria [51]. Sabry (1994) [50] reported that a shaking speed of 120 rpm is optimal for *Pseudonocardia compacta* S-39, whereas a shaking speed of 150 rpm is optimal for *Streptomyces fradiae* [14].

**Fig. 2:**
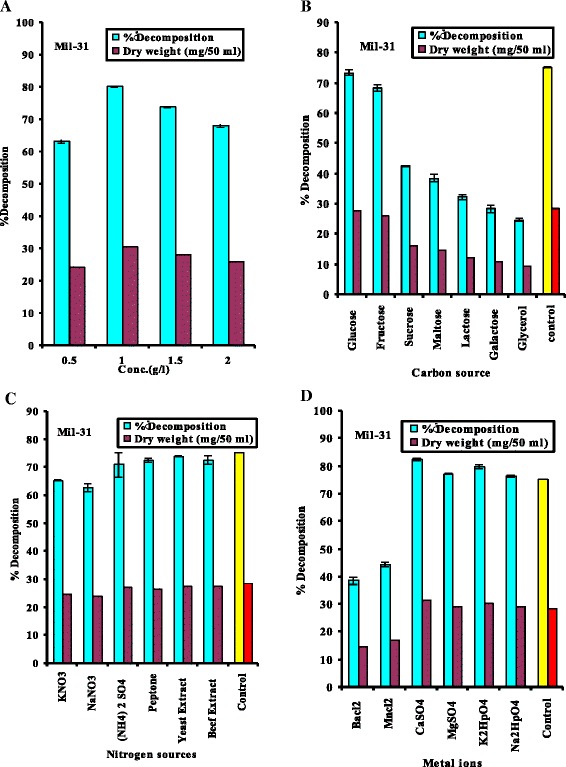
(**a**, **b**, **c** and **d**): Effect of different concentration of cholesterol, carbon sources, nitrogen sources and different metal ions on the biomass and cholesterol decomposition by *E. hirae* respectively. Difference between values is statistically significance level of 0.05

The results in Fig. 2a, show that the maximum percentage of cholesterol decomposition (80.2 %) by the *E. hirae* strain was achieved at 1 g/l cholesterol added (control sample). The present investigation detected that, the growth and decomposition of cholesterol by the tested bacterial isolates were greatly affected by cholesterol concentration in the cultural medium, whereas maximal cholesterol decomposition by *Streptomyces fradiae* (Yazdi et al., 2001) [14] and *Rhodococcus erythropolis* ATCC 25544 (Sojo et al., 2002) [24] was obtained with 2 gm/l.

The results in Fig. 2b and c show that the maximum cholesterol decomposition (75.3 %) by *E. hirae* was obtained when the growth medium lacked any additional carbon or nitrogen source (control sample). The inhibitory effect of different carbohydrates on ChoX enzyme production may be due to the decrease in bacterial ability to metabolite these carbohydrates, suggesting that, the enzyme play an important role in bacterial metabolism These results agree with those presented by [8] who reported that the *Lactobacillus*, *Streptococcus*, *Enterococcus* and *Leuconostoc* genera use cholesterol as a source of carbon. Sabry (1994) [50] observed that glycerol and xylose are the best carbon sources for the decomposition of cholesterol and glucose, and the use of starch as a carbon source resulted in a lower percentage of cholesterol decomposition by *Pseudonocardia compacta* S-39. This researcher also reported that sodium nitrate, ammonium sulphate and ammonium nitrate are the best nitrogen sources for cholesterol decomposition by *Pseudonocardia compacta* S-39.

The results in Fig. 2d, show the percentages of cholesterol decomposition achieved by each tested strain in the presence of metal ions. The results showed that highest percentage of cholesterol decomposition of 82.5 % was achieved with liquid media supplemented with 0.5 % CaSO_4_. Metal ions affecting on the growth and decomposition of cholesterol in liquid medium by *E. hirae*. In addition, the results shown in Figs. 1 and 2 show that increasing or decreasing the growth (dry weight) of *E. hirae* led to a corresponding increase or decrease in the percent of cholesterol decomposition.

The maximum percentage of cholesterol decomposition (80.4 %) by *E. hirae* was achieved using the basal medium. This result may be because the basal medium is suitable for enzyme production and activity because it contains minerals that act as a prosthetic group for the enzyme. Leland (1976) [52] recorded that the inactivation of enzyme by metals may be due to the binding of metal to the prosthetic groups of enzymes (oligodynamic effect). Sabry (1994) [50] observed that 0.04-0.1 % (w/v) of MgSO_4_ and 0.01-0.02 % (w/v) of K_2_HpO_4_ were most favourable for cholesterase production by *Pseudonocardia compacta* S-39. Gruber *et al.,* (1979) [53] found that it is possible to increase the cholesterol oxidase content of the said microorganisms several fold and at the same time to double the dry bacterial mass per liter of culture solution. The activity of cholesterol oxidase is directly and sensitively dependent on the physical properties of the membrane into the active site in which its substrate is bound [54].

**Fig. 3:**
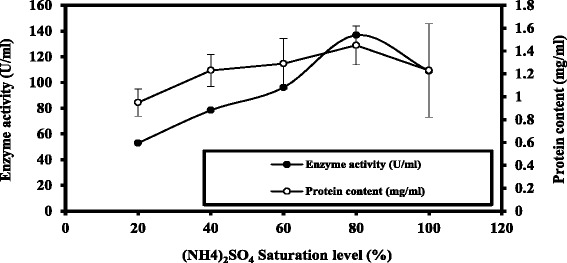
Fractional precipitation of crude cholesterol oxidase enzyme by ammonium sulphate saturation levels. The difference between values is statistically significance level of 0.05

**Fig. 4:**
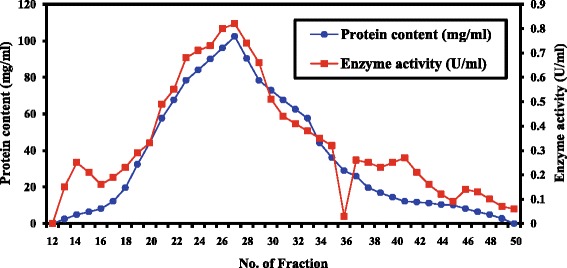
Fractional purification pattern of the dialysed cholesterol oxidase enzyme by Sephadex G-100 column chromatography

A summary of the purification data of ChoX enzyme produced by *E. hirae* is presented in Table 2 and Figs. 3 and 4. Our results showed that the partial purification of the enzyme increased the enzyme’s specific activity from 53.30 ± 0.02 to 124.87 ± 0.02 (U/mg protein), which corresponds to 2.3-fold purification and 79 % yield at the Sephadex G-100 purification step. It was shown that subsequent steps of purification program for *E. hirae* which have been carried out, the resultant precipitate was dissolved in a least amount of 10mMTris HCl buffer pH 8.0, and then it was dialysed against distilled water to exclude the sulphate ions. Furthermore it was concentrated by dialysis against sucrose crystals and consequently applied to Sephadex G-100 column chromatography. Active fractions of the sharp peak of fractional purification curve were collected and concentrated by dialysis against the same buffer for having a concentrated preparation of the purified cholesterol oxidase enzyme.

**Table 2:** Summary of purification steps

Purification steps	Enzyme activity (U/ml)	Protein content (mg/ml)	Specific enzyme activity (U/mg.protein)	Purification folds	Yield (%)
Cell free filtrate(crude)	129.60 ± 0.03	2.43 ± 0.06	53.30 ± 0.02	1.00	100
(NH_4_)_2_SO_4_ saturation level (80 %)	136.90 ± 0.05	1.45 ± 0.02	94.40 ± 0.04	1.80	105.70
Sephadex G-100	102.40 ± 0.02	0.82 ± 0.04	124.87 ± 0.02	2.30	79

Difference between values is statistically significance level of 0.05

The present study was extended for the purification and characterisation of ChoX enzyme produced by *E. hirae*. Our results are in agreement with that of [12], who recorded an optimum pH of 6.8 to 8.0 for the purified ChoX produced by *Pseudomonas* sp*.* However, [13] reported that the optimum pH value for purified ChoX activity for *Brevibacterium* sp. ranged from 5.0 to 7.5 when using 10.0 mM phosphate buffer, while 50 mM Tris–HCl buffer increased the optimum pH value to 8.0 to 8.8. In addition, the maximal production occurred at pH 6.5 for *Brevibacterium sterolicum* nov. sp. ATCC21387 [29].

Table 3 shows the maximum enzyme activity (90.2 U/ml( of the purified enzyme was achieved at 30 min. The enzyme activity was enhanced by increasing the incubation temperature up to 40 °C at which the optimum enzyme activity (84.1 U/ml) was found. The maximum cholesterol oxidase activity (89.8 U/ml) was obtained at pH 7.8 using 0.2 M Tris-HCl buffer, incubated at 40 °C for 30 min. The optimum cholesterol concentration for maximum enzyme activity (90.1 U/ml) was achieved at 1 g/l cholesterol. The maximum enzyme activity of the purified enzyme found in our study agreed with that reported by [14] for *Streptomyces fradiae*, whereas [28] noted that 1 min was optimal for *Bacillus subtilis* SFF34. The enzyme activity was enhanced by increasing the incubation temperature. However, other authors reported that the temperature of 53 °C was optimal for cholesterol oxidase activity from *Brevibacterium sp.* [13], while [14, 29] reported optimum temperatures of 70 and 55 °C for the ChoX enzyme purified from *Streptomyces fradiae* and *Brevibacterium sterolicum* nov. sp. ATCC21387, respectively. The optimum cholesterol concentration for maximum enzyme activity was in agreement with [55]. Tietz (1994) [55] reported that the rate of enzymatic reaction is generally proportional to the concentration of the enzyme. In addition to the incubation period of the enzyme and substrate, the storage time of the purified enzyme also affects ChoX enzyme activity.

**Table 3:** Effect of different incubation temperatures, pH values, substrate (cholesterol) concentrations on the cholesterol oxidase activity produced by *E. hirae* Mil−31

Incubation periods (min)	Enzyme activity (U/ml)	PH values	Enzyme activity (U/ml)	Substrate conc.(g/l)	Enzyme activity (U/ml)
10	67.90 ± 0.21	7.2	58.30 ± 0.72	0.25	67.40 ± 0.51
20	78.30 ± 0.63	7.4	67.40 ± 0.32	0.50	77.40 ± 0.21
30	90.20 ± 0.42	7.6	84.20 ± 0.36	0.75	83.50 ± 0.42
40	84.60 ± 0.52	7.8	89.80 ± 0.42	1.00	90.10 ± 1.32
50	77.40 ± 0.38	8.0	82.40 ± 0.33	1.25	85.20 ± 0.87
60	71.90 ± 0.1	8.2	77.20 ± 1.3	1.50	74.40 ± 0.4
70	66.90 ± 0.51	8.4	69.40 ± 0.45	1.75	68.20 ± 0.55
80	58.80 ± 0.4	8.6	65.80 ± 0.72	2.00	58.10 ± 1.17
90	52.60 ± 0.87	8.8	52.70 ± 0.31	-	-
-	-	9.0	43.20 ± 0.67	-	-

Difference between values is statistically significant ± LSD at probability of 0.05 level

**Table 4:** Effect of different activators and inhibitors on the cholesterol oxidase activity of the enzyme produced by *E. hirae*

Enzyme	(U/ml)			
Activity	PPM			
Metal Ions				
	Control	100	200	300
EDTA	90 ± 0.01	82 + ±0.05	78 ± 0.04	70 ± 0.03
MgCl2	90 ± 0.03	91 ± 0.06	94 ± 0.03	84 ± 0.06
CoCl2	90 ± 0.02	78 ± 0.02	68 ± 0.05	61 ± 0.05
CdCl2	90 ± 0.01	81 ± 0.04	74 ± 0.03	65 ± 0.04
KCl	90 ± 0.02	92 ± 0.03	83 ± 0.02	77 ± 0.03

Difference between values is statistically significance level of 0.05

**Fig. 5:**
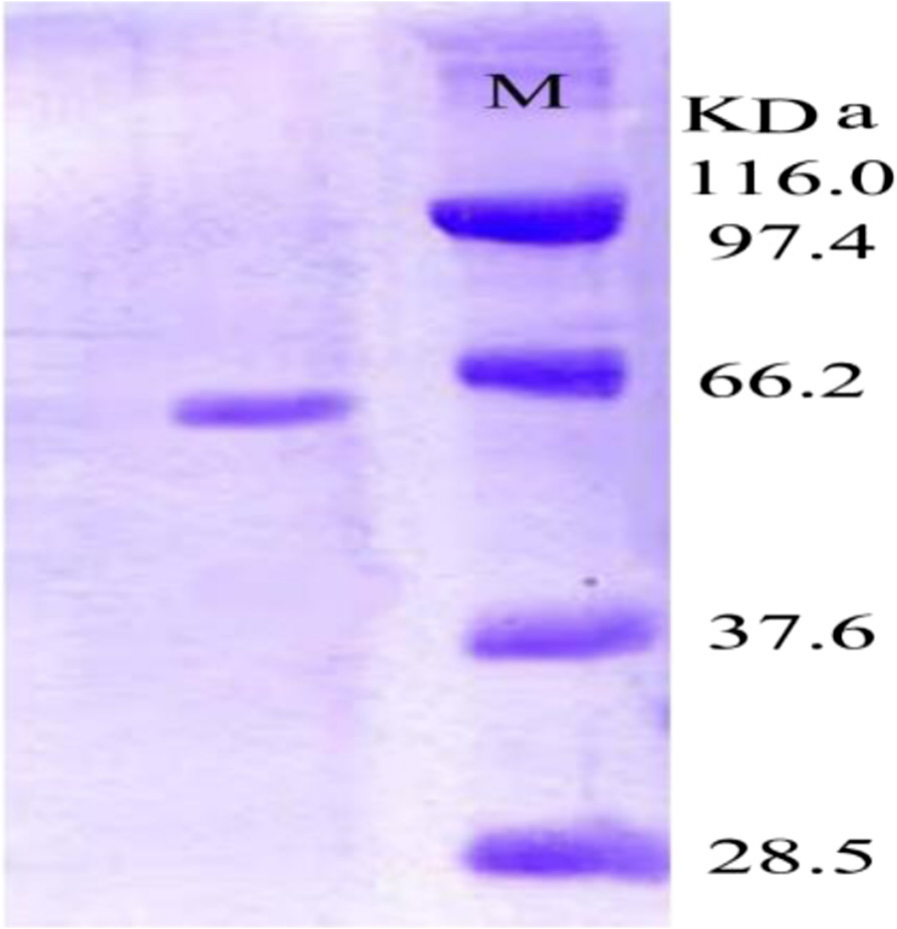
The molecular weight of the purified cholesterol oxidase enzyme produced by *Enterococcus hirae* on SDS- PAGE (15 % acrylamide) and stained with coomassie brilliant blue dye

We also found that the molecular weight of the purified cholesterol oxidase enzyme produced by *E. hirae* is 60 kDa, in comparison with the standard protein markers. as shown in Fig. 5. Moreover, different factors that affect the purified cholesterol oxidase enzyme produced by *E. hirae* were studied and indicated that the continuous increase in cholesterol oxidase activity is due to the corresponding linear increase in the enzyme concentration. The same molecular weight of ChoX enzyme purified from *Streptomyces fradia* and *Rhodococcus erythropolis* ATCC 25544 [14–24]. However, the molecular weights of ChoX enzyme purified from *Burkholderia cepacia* [17], *Streptomyces sp*. SA-COO [56] and *Gamma-proteobacterium* Y-134 [16] were 58.7, 55 and 58 kDa, respectively. This finding is in agreement with [50], who reported that the rate of an enzymatic reaction is generally proportional to the enzyme concentration. Praveen et al. (2011) [57] purified the extracellular cholesterol oxidase (cho) enzyme from *Streptomyces parvus* as a new source, and 18-fold purification was achieved by ion-exchange and gel filtration chromatography. The specific activity of the purified enzyme was found to be 20 U/mg with a 55 kDa molecular mass.

**Fig. 6:**
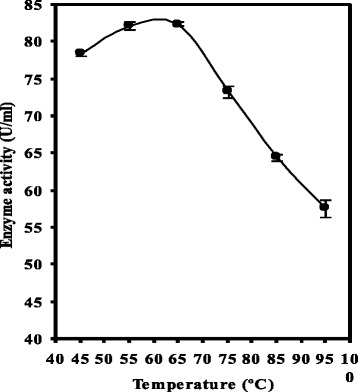
Effect of different temperature stability of the activity of the cholesterol oxidase produced by *E. hirae*. The differences between values are statistically significant ± LSD at a probability of 0.05

Regarding to thermal stability of the purified ChoX enzyme produced by *E. hirae*, the results presented in Fig. 6 show that 45–55 °C was the optimal temperature range for activity of the purified ChoX enzyme (82.4 and 82.1 U/ml(. In addition, although increasing the temperature reduced the enzyme activity, approximately 58.5 % of the COD enzyme activity was maintained at the high temperature of 95 °C. Enzyme inactivation may be due to protein denaturation at the high temperature and consequently affected the reaction rate. Salva et al. (1999) [13] found that the thermal stability for *Brevibacterium* sp. was 40 °C. Yazdi et al. (2001) [14] reported that the thermal stability of ChoX produced by *Streptomyces fradiae* was very high. ChoX had full activity at 50 °C after 90 min in 0.1 M phosphate buffer at pH 7.0. They also added that the enzyme was stable at 60 °C for 20 min. Metal ions were also observed to influence the activity of ChoX purified from *E. hirae*. Calcium alginate entrapment is a promising method for the immobilisation of *Streptomyces* sp. and also suggested that the immobilised cells could be used for three consecutive fermentation cycles for ChoX production in higher quantities compared with free cells [27]. The results obtained can be exploited for commercial purposes. In contrast, the enzyme was stable for one week when stored at 2–8 °C [50]. The enzyme activity was reduced after two weeks and continued to decrease gradually until it was completely lost at the end of 14 weeks. However, Salva *et al*. (1999) [13] reported that for *Brevibacterium* sp., the maximum enzyme activity was stable up to 15 days of storage and activity was lost at 30 days. Inactivation of the enzyme may be due to protein denaturation resulting from the high temperature, which consequently affected the rate of reaction.

Through this research the relation effect of different activators and inhibitors on the activity of the purified ChoX enzyme produced by *E. hirae* was studied. The results shown in Table 4, indicate that MgCl_2_ (200 PPM) and KCl (100 PPM) induced the maximum enzyme activity to 94.4 and 91.2 U/ml, respectively. However, EDTA, COCl_2_ and CdCl_2_ had an inhibitory effect. Leland (1976) [52] reported that the stimulatory effect of metals on enzyme activity might be related to the fact that metals act as a binding link between the enzyme and substrate or they may act as a coenzyme. Alternatively, the inhibitory effect may be related to binding of the metal to prosthetic groups of the enzyme. This finding is in agreement with [58], who observed that Mg^++^ is the best inducer for exo-enzymes at 250 μg/ml and for endo-enzymes at 100 μg/ml, while Cd^++^, Co^++^, Ba^++,^ and Ca^++^ ions were endo-enzyme inhibitors. On the other hand, Sabry (1994) [50] observed that 100 μg MgSO_4_ was the most stimulatory of the metal ions, followed by 50 μg ZnSO_4_ for the exo-enzyme produced by *Pseudonocardia compact* S-*.* It can be concluded that *E. hirae* produced ChoX enzyme and that the percent of cholesterol decomposition varied according to enzyme production conditions. Additionally, the purification and characterisation of ChoX enzyme enhanced cholesterol decomposition under certain conditions.

## Conclusions

Some species of genus *Enterococcus* which colonize the intestinal tract and reduce cholesterol level in serum. *E. hirae* Mil-31 isolated from milk had a great capacity to decompose cholesterol in basal medium supplemented with cholesterol under its optimal growth conditions (one day incubation at 37 °C, pH 7 under static condition and 1 g/l cholesterol). Decomposition process of cholesterol by this strain results from its production of cholesterol oxidase enzyme (ChoX). The highest specific enzyme activity and highest purification fold of purified enzyme were achieved after using Sephadex G-100. Also, purification and characterization of ChoX enzyme enhanced the cholesterol decomposition under certain conditions (1.45 mg.protein/ml of enzyme was incubated for 30 min at 40 °C; pH 7.8; 1 (g/l) of substrate and 200 PPM of MgCl_2_).
